# Dietary Protein Intake and Associated Risks for Atopic Dermatitis, Intrinsic Eczema, and Allergic Sensitization among Young Chinese Adults in Singapore/Malaysia: Key Findings from a Cross-sectional Study

**DOI:** 10.1016/j.xjidi.2023.100224

**Published:** 2023-08-21

**Authors:** Jun Jie Lim, Kavita Reginald, Yee-How Say, Mei Hui Liu, Fook Tim Chew

**Affiliations:** 1 Department of Biological Sciences, Faculty of Science, National University of Singapore, Singapore, Singapore; 2 Department of Biological Sciences, School of Medicine and Life Sciences, Sunway University, Selangor, Malaysia; 3 Department of Biomedical Science, Faculty of Science, Universiti Tunku Abdul Rahman (UTAR), Perak, Malaysia; 4 Department of Food Science & Technology, Faculty of Science, National University of Singapore, Singapore, Singapore

## Abstract

Through an investigator-administered questionnaire that follows the standard protocol of the International Study of Allergy and Asthma in Childhood, data on symptomatic histories of eczema and dietary habits were collected from 11,494 young Chinese adults in Singapore/Malaysia. Allergic sensitization status was assessed through a skin prick test reactivity to common house dust mites. Using three dietary indices (dietary protein score, animal protein score, and plant protein score), the associations between atopic dermatitis, intrinsic eczema, allergic sensitization, and intake of various proteins were estimated. On average, most subjects frequently eat meat, vegetables, and rice in their diets. Through a multivariable logistic regression adjusted for age, sex, body mass index, and parental eczema, subjects with high dietary protein score (adjusted OR = 1.397; 95% confidence interval = 1.133–1.724; *P* < 0.003) and high animal protein score (adjusted OR = 1.353; 95% confidence interval = 1.106–1.682; *P* < 0.003) were associated with increased risk of atopic dermatitis. Interestingly, synergy factor analysis revealed that a higher intake of plant proteins than animal proteins in diets significantly reduced overall associated risks of atopic dermatitis and allergic sensitization but not those of intrinsic eczema. Most importantly, these associations are independent of dietary fat intake. Taken together, frequent adherence to diets rich in plant proteins reduced associated risks of atopic dermatitis in Singapore/Malaysia Chinese adults.

## Introduction

Atopic dermatitis (AD) is a specific type of eczema that is marked with an elevated IgE level and associated with a personal and/or parental history of atopic diseases. This is in contrast to intrinsic eczema (IE), which has been suggested to be an inflammatory cutaneous disease that is not associated with an allergic background nor a family history of atopy (Brenninkmeijer et al., 2008; Karimkhani et al., 2015). Although AD often starts in childhood, it can persist into or develop for the first time in adulthood (Brenninkmeijer et al., 2008). In Asia, AD prevalence and its associated disease burden have been rising tremendously over the last few decades (Tsai et al., 2019). Patients with AD face a significant impact on their health-related QOL and socioeconomic well-being (Drucker et al., 2017). Despite most patients with AD suffering from similar clinical symptoms, including intense itching and recurrent inflamed rash, the severity of symptoms and underlying causes of the disease vary from person to person (Chovatiya and Silverberg, 2022). The disease pathogenesis of AD is complex and multifactorial, in which numerous synergized factors, including genetic, biological, environmental, lifestyles, and even dietary habits, interact to complicate AD development (Bauer, 2017; Brown et al., 2020; Ng and Chew, 2020). Recent findings have suggested a strong association between specific dietary patterns or nutrients and AD (Khan et al., 2022; Lim et al., 2022; Silverberg et al., 2016).

For instance, a high intake of fatty acids such as palmitoleic acid, arachidonic acid, and omega-3 fatty acids is critical in steering inflammatory responses to promote the development of allergic diseases (Venter et al., 2019). On the other hand, individuals with AD tend to practice food avoidance and eliminate certain foods in their diets to prevent further symptom aggravations (Nosrati et al., 2017). Common trigger foods for AD are thought to be mostly protein related, such as naturally occurring proteins in dairy products, eggs, peanuts, and fish (Kanny, 2005). Owing to this reason, current studies are heavily concentrated on the role of proteins as a common food allergen in worsening allergic symptoms in susceptible individuals (Domínguez et al., 2020). However, fewer studies have investigated the relationship between dietary protein intake and AD. Although there is existing evidence of an association between animal protein diets and inflammation and other chronic diseases, no studies have yet to demonstrate the association with the increased risks of allergic disorders. On the contrary, the nutritional role of plant-based protein diets in improving health outcomes is widely discussed and advocated. This includes lowering body mass index, inflammatory markers, and even the incidence of metabolic syndromes (Ahnen et al., 2019; Azzini et al., 2022; Markova et al., 2020). We have shown previously that eating vegetables and fruits frequently, as opposed to eating high-fat or high-glycaemic index (GI) foods, reduced the associated risks for severe AD (Lim et al., 2022).

At present, there are limited studies in a large adult population on the implication of AD and intake frequencies for animal- and plant-based proteins. Therefore, this study attempts to address this gap by ascertaining the association between animal- and plant-based dietary protein intake and AD. Utilizing the data obtained from the Singapore/Malaysia Cross-sectional Genetic Epidemiology Study cohort, we evaluated the intakes of animal- and plant-based protein foods in diets across young Chinese adults (aged 18–22 years). We also seek to examine how frequent intakes of these different protein sources modulate the associated risks for allergic sensitization, IE, and AD through the use of three dietary protein score (DPS) indices. These findings on various protein dietary intakes will be useful to guide future dietary interventions in patients with AD and provide valuable insights for AD-related clinical trials.

## Results

### Population demographics

**Figure 1:**
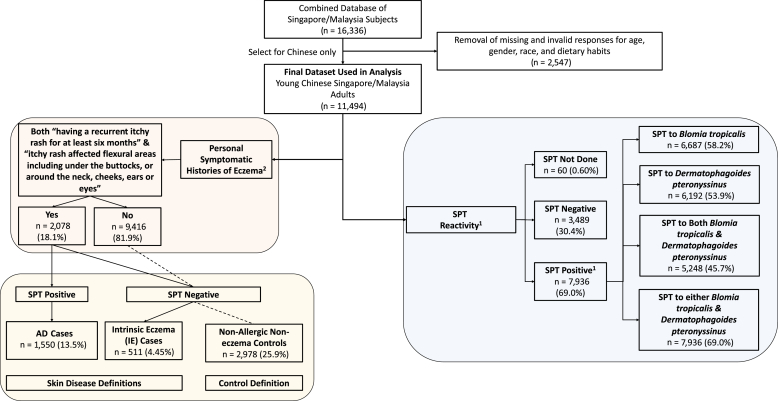
**Flowchart summarizing the classification of AD disease and allergic sensitization status among the SMCGES cohort.** AD, atopic dermatitis; SMCGES, Singapore/Malaysia Cross-sectional Genetic Epidemiology Study; SPT, skin prick test.

Among 11,494 Chinese subjects, the prevalence of AD (n = 1,550; 13.5%) was higher than the prevalence of IE (n = 511, 4.45%), and a significant proportion of subjects tested positive for skin prick test (SPT) (n = 7,936; 69.0%) (Figure 1). There was no significant difference between the mean age (range = 21.92–23.26 years) and mean body mass index (20.96–21.04 kg/m^2^) among those with IE and AD (Table 1). Most subjects have a healthy body mass index range between 18.5 and 23.0 kg/m^2^, and there was a preponderance of female subjects (>50.0%) across the two groups. Interestingly, the proportion of positive parental history of eczema in those with IE (17.1%) was similar to the proportion in those with AD (17.9%). It was previously highlighted that a parental history of eczema is a major risk factor for AD, and thus, we have adjusted it alongside age, sex, and body mass index in our multivariable logistic regression analysis to control for potential confounding effects.

**Table 1:** Demographics of 11,494 Young Chinese Adults from the SMCGES Population

Demographics	IE^1^		AD^2^	
Total Cases	511		1,550	
Age, y, mean ± SD	23.26 ± 7.12		21.92 ± 5.18	
BMI, kg/m^2^, mean ± SD	21.00 ± 3.02		21.04 ± 3.34	
	n (%)	Chi-square *P*-value^3^	n (%)	Chi-square *P*-value^3^
Sex				
Male	125 (24.5%)	5.862 × 10^−2^	669 (43.2%)	**2.200 × 10**^**−16**^
Female	386 (75.5%)		881 (56.8%)	
BMI, Asian class (kg/m^2^)				
Normal (18.5–23.0)	317 (62.0%)	5.333 × 10^−2^	914 (59.0%)	1.224 × 10^−3^
Underweight (<18.5)	57 (11.2%)		200 (12.9%)	
Overweight (>23.0)	97 (19.0%)		295 (19.0%)	
NA	40	—	141	—
Parental History of Eczema				
None	246 (48.1%)	**2.200 × 10**^**−16**^	725 (46.8%)	**2.200 × 10**^**−16**^
At least one	79 (15.5%)		251 (16.2%)	
Both	8 (1.57%)		27 (1.70%)	
NA	178	—	547	—

Abbreviations: AD, atopic dermatitis; BMI, body mass index; IE, intrinsic eczema; NA, not available; SMCGES, Singapore/Malaysia Cross-sectional Epidemiology Study; SPT, skin prick test.

1 IE case is defined to be a subject with a recurrent itchy rash in flexural areas but having a negative SPT response.

2 AD case is defined to be a subject with positive SPT to either *Blomia tropicalis* or *Dermatophagoides pteronyssinus* and the presence of a recurrent itchy rash in flexural areas.

3 Chi-square test was performed for each indicated variable for IE presentation (IE cases vs. nonallergic noneczema controls) and AD presentation (AD cases vs. nonallergic noneczema controls). Chi-square *P*-value < 0.001 (adjusted by Bonferroni’s correction) is considered statistically significant and is written in bold.

### Dietary intakes of animal and plant proteins

**Figure 2:**
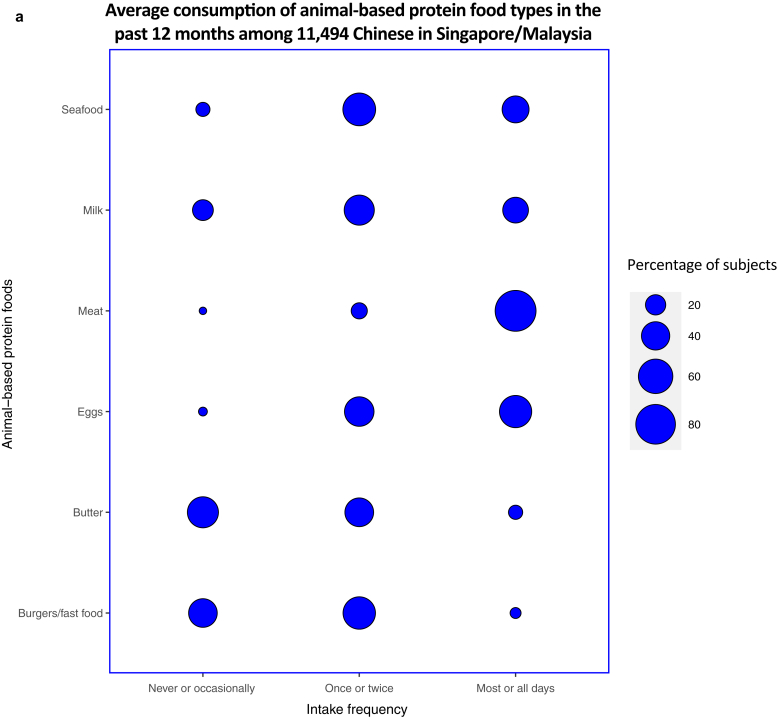

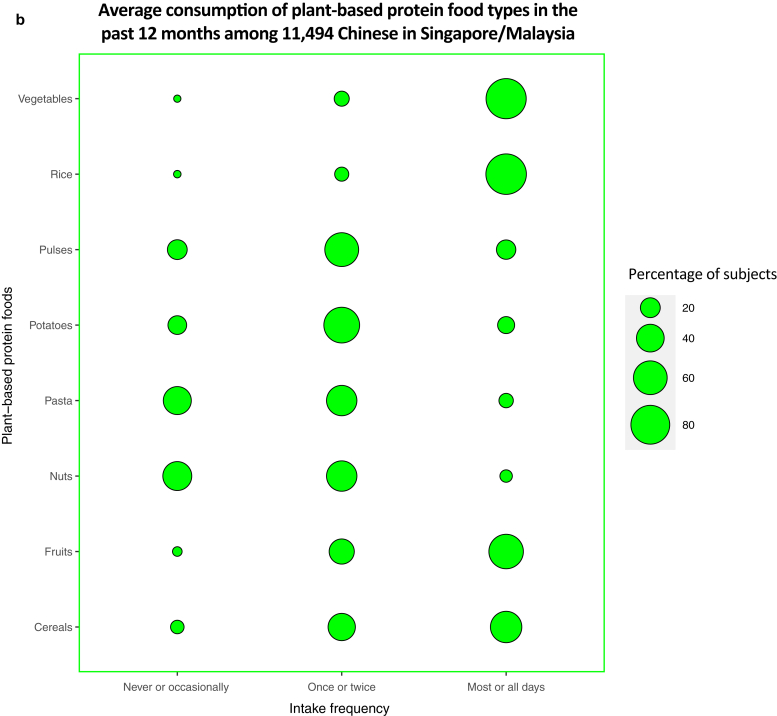
**Average consumption of selected food types in the past 12 months among the diets of 11,494 young Chinese adults from the SMCGES cohort.** The intake frequencies for each food type are represented in a balloon plot in terms of the percentage of subjects, with the food types classified on the basis of their origin source: (**a**) animal-based protein foods and (**b**) plant-based protein foods. SMCGES, Singapore/Malaysia Cross-sectional Genetic Epidemiology Study.

Most subjects (n = 9,932; 86.4%) reported meat consumption for most or all days, making meat the most consumed animal-based protein food in their diets in the past 12 months. The intake frequency of eggs on most or all days was reported to be the second highest for animal-based protein food at 53.8% (n = 6,183). Fewer subjects (at 11.5 and 6.84%, respectively) reported butter and burgers/fast food consumption frequently, making the contribution of these foods in terms of animal proteins low (Figure 2a).

Regardless of protein source, rice was reported to be the most frequently consumed food group on most or all days among subjects at 87.8% (n = 10,096) (Figure 2b). This result is not unexpected because rice forms the staple food in the diets of most Chinese individuals residing in Asia (Muthayya et al., 2014). For other plant-based protein foods, the intake frequency of vegetables on most or all days stands the highest at 83.6% (n = 9,606), followed by fruits at 59.8% (n = 6,879), and cereals at 51.1% (n = 5,868). The findings were supported by the 2018 Singapore National Nutrition Survey, which indicated an overall improvement in the dietary patterns for higher fruit and vegetable intakes among most Singaporeans (Health Promotion Board, 2018). However, intake of pasta and nuts on most or all days was comparatively lower than that of other plant-based protein foods at 10.6% (n = 1,222) and 7.99% (n = 918), respectively. Overall, the average consumption of rice, meat, and vegetables dominated the diets of our Singapore/Malaysia Cross-sectional Genetic Epidemiology Study cohort.

### Associations between various intake frequency of dietary protein and AD

We then examined the intake frequencies of various proteins, as determined by the indices, among our subjects (Table 2, Table 3, Table 4). A higher proportion of AD cases (29.5%) showed a high DPS than those of the nonallergic noneczema controls (22.0%), indicating that the intake frequency of high-protein foods in their diets, on average, was higher among patients with AD. Interestingly, there were more AD cases (37.9%) than nonallergic noneczema controls (30.4%) to have a high animal protein score (APS). However, the proportion of AD cases (33.7%) to have a high plant protein score (PPS) was slightly lower than those of nonallergic noneczema controls (35.7%). Chi-square analysis revealed that most differences between AD and nonallergic noneczema were statistically significant. Despite the trends being similar between IE cases and nonallergic noneczema controls, the differences were mostly statistically insignificant.

**Table 2:** Distribution of AD Cases and Nonallergic Noneczema Controls Based on the Various Established Dietary Indices among 11,494 Young Chinese Adults in the SMCGES

AD Presentation	AD Case (n = 1,550)	Nonallergic Noneczema Control (n = 2,978)	Chi-Square *P*-Value
DPS			
Low total protein score	508 (32.8%)	1145 (38.4%)	**6.249 × 10**^**−7**^
Moderate total protein score	584 (37.7%)	1177 (39.5%)	
High total protein score	458 (29.5%)	656 (22.0%)	
APS			
Low APS	467 (30.1%)	1041 (35.0%)	1.553 × 10^−6^
Moderate APS	495 (37.9%)	1031 (34.6%)	
High APS	588 (37.9%)	906 (30.4%)	
PPS			
Low PPS	504 (32.5%)	856 (28.7%)	3.167 × 10^−2^
Moderate PPS	523 (33.7%)	1059 (35.6%)	
High PPS	523 (33.7%)	1063 (35.7%)	
Combined animal/plant score			
High APS and low PPS	100 (6.50%)	109 (3.70%)	**6.029 × 10**^**−9**^
High APS and moderate PPS	193 (12.5%)	252 (8.50%)	
High APS and high PPS	295 (19.0%)	545 (18.3%)	
Moderate APS and low PPS	179 (11.5%)	306 (10.3%)	
Moderate APS and moderate PPS	177 (11.4%)	422 (14.2%)	
Moderate APS and high PPS	139 (9.00%)	303 (10.2%)	
Low APS and low PPS	225 (14.5%)	441 (14.8%)	
Low APS and moderate PPS	153 (9.90%)	385 (12.9%)	
Low APS and high PPS	89 (5.70%)	215 (7.20%)	
Combined dietary scores (DPS and DFS)			
High PPS and low-fat score	160	283	**6.406 × 10**^**−8**^
High PPS and moderate-fat score	212	502	
High PPS and high-fat score	178	333	
Moderate PPS and low-fat score	133	228	
Moderate PPS and moderate-fat score	151	400	
Moderate PPS and high-fat score	189	411	
Low PPS and low-fat score	183	248	
Low PPS and moderate-fat score	52	135	
Low PPS and high-fat score	292	438	
Combined dietary scores (DPS and QDGIS)			
High PPS and good QDGIS class	185	327	1.147 × 10^−1^
High PPS and moderate QDGIS class	282	566	
High PPS and poor QDGIS class	172	384	
Moderate PPS and good QDGIS class	69	113	
Moderate PPS and moderate QDGIS class	195	429	
Moderate PPS and poor QDGIS class	204	414	
Low PPS and good QDGIS class	124	216	
Low PPS and moderate QDGIS class	98	161	
Low PPS and poor QDGIS class	221	368	

Abbreviations: AD, atopic dermatitis; APS, animal protein score; DPS, dietary protein score; PPS, plant protein score; QDGIS, quality of diet based on glycaemic index score; SMCGES, Singapore/Malaysia Cross-sectional Genetic Epidemiology Study.

A chi-square *P*-value < 0.001 (as adjusted by Bonferroni’s correction for multiple comparisons) is considered statistically significant and is written in bold.

**Table 3:** Distribution of IE Cases and Nonallergic Noneczema Controls Based on the Various Established Dietary Indices among 11,494 Young Chinese Adults in the SMCGES Cohort

IE Presentation	IE Case (n = 511)	Nonallergic Noneczema Control (n = 2,978)	Chi-Square *P*-Value
DQDPS			
Low total protein score	150 (29.4%)	841 (28.2%)	5.308 × 10^−1^
Moderate total protein score	176 (34.4%)	1103 (37.0%)	
High total protein score	185 (36.2%)	1034 (34.7%)	
APS			
Low APS	182 (35.6%)	1041 (35.0%)	8.786 × 10^−1^
Moderate APS	158 (30.9%)	906 (30.4%)	
High APS	171 (33.5%)	1031 (34.6%)	
PPS			
Low PPS	157 (30.7%)	856 (28.7%)	2.721 × 10^−1^
Moderate PPS	163 (31.9%)	1059 (35.6%)	
High PPS	191 (37.4%)	1063 (35.7%)	
Combined APS/PPS			
High PPS and low APS	44 (8.60 %)	215 (7.20 %)	8.106 × 10^−2^
High PPS and moderate APS	65 (12.7%)	303 (10.2%)	
High PPS and high APS	82 (16.0%)	545 (18.3%)	
Moderate PPS and low APS	53 (10.4%)	385 (12.9%)	
Moderate PPS and moderate APS	60 (11.7%)	422 (14.2%)	
Moderate PPS and high APS	50 (9.80%)	252 (8.50%)	
Low PPS and low APS	85 (16.6%)	441 (14.8%)	
Low PPS and moderate APS	46 (9.00%)	306 (10.3%)	
Low PPS and high APS	26 (5.10%)	109 (3.70%)	
Combined dietary scores (DPS and DFS)			
High PPS and low-fat score	103 (20.2%)	502 (16.9%)	3.815 × 10^−1^
High PPS and moderate-fat score	51 (10.0%)	333 (11.2%)	
High PPS and high-fat score	37 (7.20%)	228 (7.70%)	
Moderate PPS and low-fat score	58 (11.4%)	400 (13.4%)	
Moderate PPS and moderate-fat score	57 (11.2%)	411 (13.8%)	
Moderate PPS and high-fat score	48 (9.40%)	248 (8.30%)	
Low PPS and low-fat score	26 (5.10%)	135 (4.50%)	
Low PPS and moderate-fat score	54 (10.6%)	283 (9.50%)	
Low PPS and high-fat score	77 (15.1%)	438 (14.7%)	
Combined dietary scores (DPS and QDGIS)			
High PPS and good QDGIS class	116 (22.7%)	566 (19.0 %)	6.260 × 10^−2^
High PPS and moderate QDGIS class	53 (10.4%)	384 (12.9%)	
High PPS and poor QDGIS class	22 (4.30%)	113 (3.80%)	
Moderate PPS and good QDGIS class	78 (15.3%)	429 (14.4%)	
Moderate PPS and moderate QDGIS class	56 (11.0%)	414 (13.9%)	
Moderate PPS and poor QDGIS class	29 (5.70%)	216 (7.30%)	
Low PPS and good QDGIS class	21 (4.10%)	161 (5.40%)	
Low PPS and moderate QDGIS class	71 (13.9%)	327 (11.0%)	
Low PPS and poor QDGIS class	65 (12.7%)	368 (12.4%)	

Abbreviations: APS, animal protein score; DPS, dietary protein score; DQDPS, diet quality based on dietary protein score; IE, intrinsic eczema; PPS, plant protein score; QDGIS, quality of diet based on glycaemic index score; SMCGES, Singapore/Malaysia Cross-sectional Genetic Epidemiology Study.

A chi-square *P*-value < 0.001 (as adjusted by Bonferroni’s correction for multiple comparisons) is considered statistically significant.

**Table 4:** Distribution of Positive and Negative SPT Reactivity Based on the Various Established Dietary Indices among 11,494 Young Chinese Adults in the SMCGES Cohort

SPT Reactivity to Common Mite Allergens	Positive SPT Response (n = 7,936)	Negative SPT Response (n = 3,489)	Chi-Square *P*-Value
DQDPS			
Low total protein score	1777 (22.4%)	991 (28.4%)	**4.650 × 10**^**−14**^
Moderate total protein score	2879 (36.3%)	1279 (36.7%)	
High total protein score	3280 (41.3 %)	1219 (34.9%)	
APS			
Low APS	2341 (29.5%)	1223 (35.1%)	**1.224 × 10**^**−12**^
Moderate APS	2944 (37.1%)	1064 (30.5%)	
High APS	2651 (41.3%)	1202 (34.5%)	
PPS			
Low PPS	2513 (31.7%)	1013 (29.0%)	1.703 × 10^−2^
Moderate PPS	2642 (33.3%)	1222 (35.0%)	
High PPS	2781 (35.0%)	1254 (35.9%)	
Combined APS/PPS			
High APS and low PPS	429 (5.40%)	135 (3.90%)	**3.023 × 10**^**−16**^
High APS and moderate APS	924 (11.6%)	302 (8.70%)	
High APS and high PPS	1591 (20.0%)	627 (18.0%)	
Moderate APS and low PPS	904 (11.4%)	352 (10.1%)	
Moderate APS and moderate PPS	964 (12.1%)	482 (13.8%)	
Moderate APS and high PPS	783 (9.90%)	368 (10.5%)	
Low APS and low PPS	1180 (14.9%)	526 (15.1%)	
Low APS and moderate PPS	754 (9.50%)	438 (12.6%)	
Low APS and high PPS	407 (5.10%)	259 (7.40%)	
Combined dietary scores (DPS and DFS)			
High PPS and low-fat score	1129 (14.2%)	605 (17.3%)	**1.110 × 10**^**−14**^
High PPS and moderate-fat score	940 (11.8%)	384 (11.0%)	
High PPS and high-fat score	712 (9.00%)	265 (7.60%)	
Moderate PPS and low-fat score	770 (9.70%)	458 (13.1%)	
Moderate PPS and moderate-fat score	994 (12.5%)	468 (13.4%)	
Moderate PPS and high-fat score	878 (11.1%)	296 (8.50%)	
Low PPS and low-fat score	301 (3.80%)	161 (4.60%)	
Low PPS and moderate-fat score	806 (10.2%)	337 (9.70%)	
Low PPS and high-fat score	1406 (17.7%)	515 (14.8%)	
Combined dietary scores (DPS and QDGIS)			
High PPS and good QDGIS class	1446 (18.2%)	682 (19.5%)	**4.569 × 10**^**−4**^
High PPS and moderate QDGIS class	996 (12.6%)	437 (12.5%)	
High PPS and poor QDGIS class	339 (4.30%)	135 (3.90%)	
Moderate PPS and good QDGIS class	943 (11.9%)	507 (14.5%)	
Moderate PPS and moderate QDGIS class	1049 (13.2%)	470 (13.5%)	
Moderate PPS and poor QDGIS class	650 (8.20%)	245 (7.00%)	
Low PPS and good QDGIS class	453 (5.70%)	182 (5.20%)	
Low PPS and moderate QDGIS class	931 (11.7%)	398 (11.4%)	
Low PPS and poor QDGIS class	1129 (14.2%)	433 (12.4%)	

Abbreviations: APS, animal protein score; DPS, dietary protein score; DQDPS, diet quality based on dietary protein score; PPS, plant protein score; QDGIS, quality of diet based on glycaemic index score; SMCGES, Singapore/Malaysia Cross-sectional Genetic Epidemiology Study; SPT, skin prick test.

A chi-square *P*-value < 0.001 (as adjusted by Bonferroni’s correction for multiple comparisons) is considered statistically significant and is written in bold.

In both univariable and multivariable models, we confirmed that a frequent intake of high-protein foods in diets was only significantly associated with an increased risk of AD and allergic sensitization but not of IE (Table 5). Furthermore, there was a clear dose-dependent increase in the ORs and adjusted ORs (AORs) from a moderate to high DPS for AD and allergic sensitization. Thus, this altogether suggests a strong association between an intake frequency for high-protein foods and disease outcomes, and the association was largely dependent on the subjects’ allergic background. This was consistent with the associations of APS and PPS. For allergic sensitization, higher APSs were positively associated with allergic sensitization (AOR = 1.297) and AD (AOR = 1.353). Meanwhile, the association between having a high PPS, AD, and allergic sensitization was marginally insignificant in the multivariable model.

**Table 5:** Association between Three Protein Dietary Indices (DPS, APS, and PPS) with IE, Allergic Sensitization, and AD Presentation among 11,494 Young Chinese adults from SMCGES Population

Dietary Scores	IE Presentation^1^			Allergic Sensitization to HDMs^2^			AD Presentation^3^		
	OR	95% CI	*P*-Value^4^	OR	95% CI	*P*-Value^4^	OR	95% CI	*P*-Value^4^
DPS, unadjusted									
Low total protein score	1.000	REF	—	1.000	REF	—	1.000	REF	—
Moderate total protein score	0.895	0.707–1.133	0.354	1.255	1.134–1.390	**1.210×10^−^^5^**	1.130	0.962–1.328	0.137
High total protein score	1.003	0.794–1.268	0.979	1.501	1.355–1.661	**5.510×10^−^^15^**	1.493	1.276–1.749	**6.130×10^−^^7^**
DPS, adjusted									
Low total protein score	1.000	REF	—	1.000	REF	—	1.000	REF	—
Moderate total protein score	0.940	0.692–1.277	0.689	1.205	1.058–1.373	0.005	1.136	0.918–1.406	0.242
High total protein score	1.036	0.771–1.421	0.775	1.409	1.236–1.606	**5.060×10^−^^3^**	1.397	1.133–1.724	**0.002**
APS, unadjusted									
Low animal protein score	1.000	REF	—	1.000	REF	—	1.000	REF	—
Moderate animal protein score	0.949	0.757–1.189	0.649	1.152	1.046–1.270	0.004	1.070	0.918–1.247	0.384
High animal protein score	0.997	0.791–1.256	0.983	1.446	1.310–1.595	**2.240×10^−^^13^**	1.447	1.245–1.682	**1.550×10^−^^6^**
APS, adjusted									
Low animal protein score	1.000	REF	—	1.000	REF	—	1.000	REF	—
Moderate animal protein score	1.000	0.743–1.346	0.998	1.116	0.984–1.266	0.088	1.010	0.821–1.242	0.925
High animal protein score	1.048	0.773–1.420	0.761	1.297	1.142–1.474	**6.540×10^−^^5^**	1.353	1.106–1.655	**0.003**
PPS, unadjusted									
Low plant protein score	1.000	REF	—	1.000	REF	—	1.000	REF	—
Moderate plant protein score	0.839	0.662–1.064	0.147	0.872	0.789–0.963	0.007	0.839	0.721–0.976	0.023
High plant protein score	0.980	0.779–1.233	0.861	0.894	0.810–0.987	0.026	0.836	0.718–0.973	0.021
PPS, adjusted									
Low plant protein score	1.000	REF	—	1.000	REF	—	1.000	REF	—
Moderate plant protein score	0.755	0.549–1.039	0.084	0.856	0.752–0.975	0.020	0.830	0.676–1.020	0.076
High plant protein score	1.025	0.760–1.385	0.873	0.874	0.768–0.994	0.041	0.822	0.669–1.010	0.062

Abbreviations: AD, atopic dermatitis; APS, animal protein score; BMI, body mass index; CI, confidence interval; DPS, dietary protein score; HDM, house dust mite; IE, intrinsic eczema; PPS, plant protein score; REF, reference; SMCGES, Singapore/Malaysia Cross-sectional Genetic Epidemiology Study; SPT, skin prick test.

For results analyzed through multivariable logistic regression, the model adjusted for age, sex, BMI, and parental eczema, as confounding factors.

1 IE subject was defined to be positive for eczema symptoms if having a recurrent itchy rash that was coming and going for at least 6 months in flexural areas but testing negative for SPT reactivity to either one of the two common HDMs.

2 Allergic sensitization was compared between SPT-positive and SPT-negative subjects. SPT-positive subjects were determined by their reactivity to either one of the two common HDMs (*Blomia tropicalis* and *Dermatophagoides pteronyssinus*).

3 AD presentation was compared between an AD case and a nonallergic noneczema control. AD was defined in this study to be affirmative to positive SPT and having eczema symptoms.

4 Logistic regression *P*-value < 0.003 (after Bonferroni’s correction for multiple comparisons) is statistically significant and written in bold.

### Antagonistic interactions between animal and plant protein in diets

**Figure 3:**
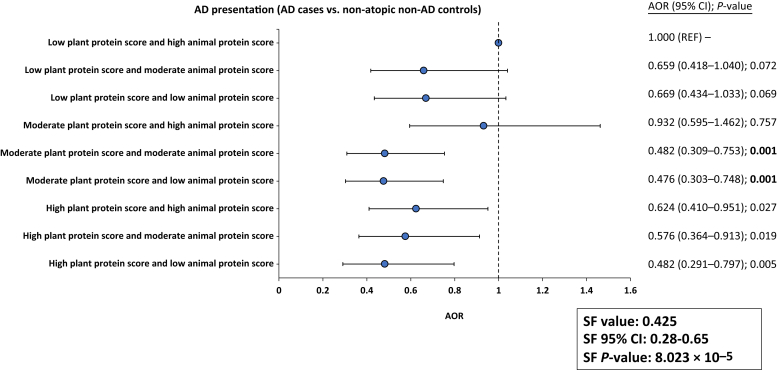
**Results obtained from the multivariable logistic regression analysis of protein dietary pattern in terms of the combined protein scores (APS and PPS) in relation to AD presentation (nonallergic noneczema controls vs. AD cases).** Age, sex, BMI, and parental eczema were adjusted for in the model, with the values presented in AOR, 95% CI, and *P*-value. *P*-values < 0.003 are statistically significant (adjusted by Bonferroni’s correction). A reference dotted line is drawn at the interception point where AOR equals 1.000. The 95% CI for each combined protein class is represented by a single line that cuts the AOR. An SF analysis revealed that APS and PPS in diets interact in an antagonistic manner to reduce the associated odds of AD (SF value < 1.000). AD, atopic dermatitis; AOR, adjusted OR; APS, animal protein score; BMI, body mass index; CI, confidence interval; REF, reference; PPS, plant protein score; SF, synergy factor.

There was clear initial evidence that an increased intake frequency of total proteins (AOR = 1.397), particularly animal-based protein foods (AOR = 1.353), was associated with increased odds for AD. However, most individuals maintain a typical diet that consists of both animal- and plant-based protein foods. Therefore, this study adopted a combined analysis with APS and PPS to understand the associations between intake frequencies of both animal- and plant-based protein foods in diets and AD. In the adjusted logistic model, there was an apparent dose–effect relationship between APS and PPS. Having a moderate plant-based protein with moderate animal-based proteins in diets was sufficient to confer a strong, significant protective association on AD (AOR = 0.482) (Figure 3). However, this was not observed for IE (Table 6, Table 7, Table 8, Table 9, Table 10, Table 11, Table 12, Table 13, Table 14, Table 15).

**Table 6:** Multivariable Logistic Regression Analysis for Intrinsic Eczema and Allergic Sensitization Using Combined Protein Scores for PPS and APS

Combined Protein Scores (PPS and APS)	Multivariable Logistic Regression (Adjusted for Age, Sex, BMI, and Parental Eczema)					
	Intrinsic Eczema			Allergic Sensitization		
	AOR	95% CI	*P*-Value^1^	AOR	95% CI	*P*-Value^1^
High PPS and low APS	0.953	0.478–1.965	0.894	0.570	0.412–0.786	**6.660×10^−^^4^**
High PPS and moderate APS	1.093	0.544–2.070	0.911	0.756	0.558–1.018	0.068
High PPS and high APS	0.663	0.354–1.302	0.214	0.795	0.598–1.049	0.110
Moderate PPS and low APS	0.518	0.262–1.056	0.062	0.586	0.434–0.785	**3.960×10^−^^4^**
Moderate PPS and moderate APS	0.528	0.268–1.076	0.070	0.686	0.510–0.915	0.011
Moderate PPS and high APS	0.912	0.460–1.868	0.794	0.985	0.724–1.333	0.924
Low PPS and low APS	0.835	0.441–1.654	0.590	0.810	0.604–1.078	0.153
Low PPS and moderate APS	0.702	0.352–1.445	0.323	0.834	0.615–1.125	0.240
Low PPS and high APS	1.000	REF	—	1.000	REF	—

Abbreviations: AOR, adjusted OR; APS, animal protein score; BMI, body mass index; CI, confidence interval; PPS, plant protein score; REF, reference.

1 *P* < 0.003 (after Bonferroni correction for multiple comparisons) are statistically significant and written in bold.

**Table 7:** SF Analysis for Intrinsic Eczema and Allergic Sensitization Using Combined Protein Scores for PPS and APS

Combined Protein Scores (PPS and APS)	SF Analysis							
	Intrinsic Eczema				Allergic Sensitization			
	OR	SF Value	SF 95% CI	SF *P*-Value	OR	SF Value	SF 95% CI	SF *P*-Value
Low PPS and low APS	1.000	1.089	0.581–2.044	7.90 × 10^−1^	1.000	0.437	0.329–0.580	**1.158 × 10**^**−8**^
Low PPS and high APS	1.238				1.417			
High PPS and high APS	0.781				1.131			
High PPS and low APS	1.062				0.700			

Abbreviations: APS, animal protein score; CI, confidence interval; PPS, plant protein score; SF, synergy factor.

*P* < 0.003 (after Bonferroni correction for multiple comparisons) are statistically significant and written in bold.

**Table 8:** Multivariable Logistic Regression Analysis for Intrinsic Eczema, Allergic Sensitization, and Atopic Dermatitis Using PPS and DQDFS

Combined Dietary Scores (PPS and DQDFS)	Multivariable Logistic Regression (Adjusted for Age, Sex, BMI, and Parental Eczema)								
	Intrinsic Eczema			Allergic Sensitization			Atopic Dermatitis		
	AOR	95% CI	*P*-Value^1^	AOR	95% CI	*P*-Value^1^	AOR	95% CI	*P*-Value^1^
High PPS and low-fat score	1.188	0.787–1.806	0.416	0.762	0.635–0.916	0.004	0.639	0.473–0.862	**0.003**
High PPS and moderate-fat score	0.932	0.575–1.500	0.772	0.831	0.680–1.016	0.070	0.821	0.600–1.122	0.218
High PPS and high-fat score	0.785	0.435–1.372	0.406	0.969	0.775–1.215	0.786	0.881	0.619–1.250	0.480
Moderate PPS and low-fat score	0.663	0.403–1.079	0.101	0.659	0.540–0.804	**4.120×10^−^^5^**	0.626	0.453–0.860	0.004
Moderate PPS and moderate-fat score	0.655	0.393–1.076	0.098	0.803	0.661–0.976	0.028	0.643	0.469–0.879	0.006
Moderate PPS and high-fat score	1.062	0.633–1.760	0.818	1.050	0.848–1.302	0.656	1.144	0.829–1.577	0.414
Low PPS and low-fat score	1.163	0.608–2.139	0.637	0.777	0.584–1.036	0.084	0.727	0.410–1.152	0.182
Low PPS and moderate-fat score	0.911	0.536–1.525	0.727	0.942	0.760–1.169	0.587	0.867	0.619–1.211	0.405
Low PPS and high-fat score	1.000	REF	—	1.000	REF	—	1.000	REF	—

Abbreviations: AOR, adjusted OR; BMI, body mass index; CI, confidence interval; DQDFS, diet quality based on dietary fat score; PPS, plant protein score; REF, reference.

1 *P* < 0.003 (after Bonferroni correction for multiple comparisons) are statistically significant and written in bold.

**Table 9:** SF Analysis for Intrinsic Eczema, Allergic Sensitization, and Atopic Dermatitis Using PPS and DQDFS

SF Analysis												
Combined Protein Scores (PPS and DQDFS)	Intrinsic eczema				Allergic Sensitization				Atopic dermatitis			
	OR	SF Value	SF 95% CI	SF *P*-Value	OR	SF Value	SF 95% CI	SF *P*-Value	OR	SF Value	SF 95% CI	SF *P*-Value
Low PPS and low-fat score	1.000	1.385	0.736–2.608	3.130 × 10^−1^	1.000	0.476	0.361–0.627	**1.384 × 10**^**−7**^	1.000	0.418	0.269–0.651	**1.129 × 10**^**−4**^
Low PPS and high-fat score	0.913				1.460				1.731			
High PPS and high-fat score	0.843				1.437				1.514			
High PPS and low-fat score	1.065				0.998				1.096			

Abbreviations: CI, confidence interval; DQDFS, diet quality based on dietary fat score; PPS, plant protein score; SF, synergy factor.

*P* < 0.003 (after Bonferroni correction for multiple comparisons) are statistically significant and written in bold.

**Table 10:** Multivariable Logistic Regression Analysis for Intrinsic Eczema, Allergic Sensitization, and Atopic Dermatitis Using PPS and QDGIS

Combined Dietary Scores (PPS and QDGIS)	Multivariable Logistic Regression (Adjusted for Age, Sex, BMI, and Parental Eczema)								
	Intrinsic Eczema			Allergic Sensitization			Atopic Dermatitis		
	AOR	95% CI	*P*-Value^1^	AOR	95% CI	*P*-Value^1^	AOR	95% CI	*P*-Value^1^
High PPS and good QDGIS class	1.265	0.818–1.991	0.299	0.856	0.708–1.034	0.108	0.856	0.634–1.156	0.308
High PPS and moderate QDGIS class	0.835	0.493–1.411	0.490	0.878	0.712–1.083	0.225	0.709	0.505–0.992	0.045
High PPS and poor QDGIS class	1.058	0.517–2.072	0.873	0.898	0.670–1.209	0.476	0.897	0.558–1.426	0.650
Moderate PPS and good QDGIS class	0.960	0.596–1.562	0.869	0.717	0.585–0.878	**0.001**	0.719	0.520–0.993	0.045
Moderate PPS and moderate QDGIS class	0.661	0.381–1.137	0.136	0.907	0.738–1.114	0.352	0.880	0.640–1.212	0.434
Moderate PPS and poor QDGIS class	0.724	0.367–1.370	0.333	1.055	0.825–1.354	0.669	0.905	0.613–1.332	0.615
Low PPS and good QDGIS class	0.914	0.460–1.746	0.791	1.044	0.797v1.371	0.756	1.063	0.698–1.611	0.773
Low PPS and moderate QDGIS class	1.211	0.733–2.010	0.455	0.964	0.779–1.195	0.741	0.930	0.664–1.302	0.671
Low PPS and poor QDGIS class	1.000	REF	—	1.000	REF	—	1.000	REF	—

Abbreviations: AOR, adjusted OR; BMI, body mass index; CI, confidence interval; QDGIS, quality of diet based on glycemic index score; PPS, plant protein score; REF, reference.

1 *P* < 0.003 (after Bonferroni correction for multiple comparisons) are statistically significant and written in bold.

**Table 11:** SF Analysis for Intrinsic Eczema, Allergic Sensitization, and Atopic Dermatitis Using PPS and QDGIS

SF Analysis												
Combined Dietary Scores (PPS and QDGIS)	Intrinsic Eczema				Allergic Sensitization				Atopic Dermatitis			
	OR	SF Value	SF 95% CI	SF *P*-Value	OR	SF Value	SF 95% CI	SF *P*-Value	OR	SF Value	SF 95% CI	SF *P*-Value
Low PPS and poor QDGIS class	1.000	1.426	0.691–2.942	3.374 × 10^−1^	1.000	0.806	0.597–1.088	1.587 × 10^−1^	1.000	0.641	0.415–0.990	**4.478 × 10**^**−2**^
Low PPS and good QDGIS class	0.738				1.048				0.987			
High PPS and poor QDGIS class	1.102				1.009				1.294			
High PPS and good QDGIS class	1.160				0.852				0.819			

Abbreviations: CI, confidence interval; PPS, plant protein score; QDGIS, quality of diet based on glycemic index score; SF, synergy factor.

*P* < 0.003 (after Bonferroni correction for multiple comparisons) are statistically significant and written in bold.

**Table 12:** Multivariable Logistic Analysis for Intrinsic Eczema, Allergic Sensitization, and Atopic Dermatitis Using APS and DQDFS

Combined Dietary Scores (APS and DQDFS)	Multivariable Logistic Regression (Adjusted for Age, Sex, BMI, and Parental Eczema)								
	Intrinsic Eczema			Allergic Sensitization			Atopic Dermatitis		
	AOR	95% CI	*P*-Value^1^	AOR	95% CI	*P*-Value^1^	AOR	95% CI	*P*-Value^1^
Low APS and low-fat score	0.884	0.602–1.300	0.529	0.624	0.529–0.735	**1.940×10^−^^8^**	0.588	0.454–0.761	**5.520×10^−^^5^**
Low APS and moderate-fat score	0.828	0.486–1.374	0.476	0.868	0.705–1.071	0.185	0.685	0.491–0.949	0.024
Low APS and high-fat score	1.156	0.587–2.153	0.659	0.794	0.600–1.057	0.110	0.804	0.516–1.237	0.326
Moderate APS and low-fat score	1.185	0.761–1.835	0.448	0.769	0.632–0.935	0.008	0.567	0.408–0.784	**6.620×10^−^^4^**
Moderate APS and moderate-fat score	0.763	0.487–1.183	0.230	0.744	0.623–0.890	**0.001**	0.649	0.491–0.856	**0.002**
Moderate APS and high-fat score	0.777	0.448–1.307	0.354	0.886	0.720–1.093	0.257	0.732	0.524–1.015	0.063
High APS and low-fat score	0.824	0.324–1.832	0.658	0.755	0.533–1.082	0.120	0.488	0.254–0.890	0.024
High APS and moderate-fat score	0.871	0.533–1.398	0.573	0.805	0.662–0.980	**0.030**	0.718	0.528–0.972	0.033
High APS and high-fat score	1.000	REF	—	1.000	REF	—	1.000	REF	—

Abbreviations: AOR, adjusted OR; APS, animal protein score; BMI, body mass index; DQDFS, diet quality based on dietary fat score; CI, confidence interval; REF, reference.

1 *P* < 0.003 (after Bonferroni correction for multiple comparisons) are statistically significant and written in bold.

**Table 13:** SF Analysis for Intrinsic Eczema, Allergic Sensitization, and Atopic Dermatitis Using APS and DQDFS

SF Analysis													
	Intrinsic Eczema				Allergic Sensitization					Atopic Dermatitis			
Combined Dietary Scores (APS and DQDFS)	OR	SF Value	SF 95% CI	SF *P*-Value	OR	SF Value	SF 95% CI	SF *P*-Value	OR		SF Value	SF 95% CI	SF *P*-Value
Low APS and low-fat score	1.000	1.106	0.489–2.498	8.094 × 10^−1^	1.000	1.002	0.708–1.420	9.902 × 10^−1^	1.000		1.200	0.697–2.066	5.113 × 10^−1^
Low APS and high-fat score	1.086				1.430				1.335				
High APS and low-fat score	0.982				1.267				1.175				
High APS and high-fat score	1.179				1.816				1.882				

Abbreviations: APS, animal protein score; CI, confidence interval; DQDFS, diet quality based on dietary fat score; SF, synergy factor.

**Table 14:** Multivariable Logistic Regression Analysis for Intrinsic Eczema, Allergic Sensitization, and Atopic Dermatitis Using APS and QDGIS

Combined Dietary Scores (APS and QDGIS)	Multivariable Logistic Regression (Adjusted for Age, Sex, BMI, and Parental Eczema)								
	Intrinsic Eczema			Allergic Sensitization			Atopic Dermatitis		
	AOR	95% CI	*P*-Value^1^	AOR	95% CI	*P*-Value^1^	AOR	95% CI	*P*-Value^1^
Low APS and good QDGIS class	1.371	0.715–2.761	0.357	0.638	0.482–0.840	**0.002**	0.624	0.408–0.955	**0.030**
Low APS and moderate QDGIS class	0.922	0.484–1.848	0.812	0.687	0.528–0.890	0.005	0.612	0.414–0.908	0.014
Low APS and poor QDGIS class	0.964	0.484–1.994	0.919	0.714	0.540–0.941	0.017	0.619	0.405–0.944	0.026
Moderate APS and good QDGIS class	1.162	0.619–2.305	0.652	0.624	0.478–0.810	**4.560×10^−^^4^**	0.542	0.362–0.812	**0.003**
Moderate APS and moderate QDGIS class	0.928	0.478–1.884	0.830	0.797	0.611–1.035	0.091	0.616	0.413–0.921	0.018
Moderate APS and poor QDGIS class	1.109	0.538–2.352	0.782	0.950	0.712–1.266	0.730	0.792	0.512–1.226	0.296
High APS and good QDGIS class	1.152	0.621–2.262	0.666	0.825	0.638–1.060	0.137	0.789	0.542–1.154	0.219
High APS and moderate QDGIS class	1.108	0.557–2.289	0.776	0.924	0.701–1.214	0.573	0.826	0.550–1.242	0.356
High APS and poor QDGIS class	1.000	REF	—	1.000	REF	—	1.000	REF	—

Abbreviations: AOR, adjusted OR; APS, animal protein score; BMI, body mass index; CI, confidence interval; QDGIS, quality of diet based on glycemic index score; REF, reference.

1 *P* < 0.003 (after Bonferroni correction for multiple comparisons) are statistically significant and written in bold.

**Table 15:** SF Analysis for Intrinsic Eczema, Allergic Sensitization, and Atopic Dermatitis Using APS and QDGIS

SF Analysis												
Combined Dietary Scores (APS and QDGIS)	Intrinsic Eczema				Allergic Sensitization				Atopic Dermatitis			
	OR	SF Value	SF 95% CI	SF *P*-Value	OR	SF Value	SF 95% CI	SF *P*-Value	OR	SF Value	SF 95% CI	SF *P*-Value
Low APS and poor QDGIS class	1.000	0.739	0.390–1.399	3.353 × 10^−1^	1.000	0.889	0.680–1.165	3.952 ×10^−1^	1.000	0.605	0.404–0.907	**1.489 × 10**^**−2**^
Low PPS and good QDGIS class	1.377				0.810				1.104			
High PPS and poor QDGIS class	1.057				1.646				1.992			
High APS and good QDGIS class	1.076				1.186				1.332			

Abbreviations: APS, animal protein score; CI, confidence interval; QDGIS, quality of diet based on glycemic index score; SF, synergy factor.

*P* < 0.003 (after Bonferroni correction for multiple comparisons) are statistically significant and written in bold.

On the basis of the synergy factor (SF) analysis, there was an antagonistic interaction between APS and PPS to lower the risks of AD (Figure 3). This supported the earlier results that despite frequent intake of high-protein foods, particularly animal-based protein foods, the increased intake of plant-based protein foods in diets nullified the overall increased associated risks of AD. As previously reported, frequent intakes of high-fat and/or high-GI foods were positively associated with AD (Lim et al., 2022). Through an SF analysis using the combined dietary scores for fats and GI with PPS, a higher intake frequency of plant-based proteins in diets was also shown to significantly reduce the odds of AD imposed by high fat and high GI (Table 6, Table 7, Table 8, Table 9, Table 10, Table 11, Table 12, Table 13, Table 14, Table 15). Interestingly, a high animal protein diet was dependent on high-fat diets in terms of the associated AD risks (Table 6, Table 7, Table 8, Table 9, Table 10, Table 11, Table 12, Table 13, Table 14, Table 15).

## Discussion

To the best of our knowledge, little is known about whether intake frequencies of different dietary protein sources could be associated with AD and potentially affect AD development through the modulation of various proinflammatory processes. This study found that a frequent intake of high-protein foods in diets, especially animal-based proteins, posed an increased associated risk of AD and allergic sensitization to common house dust mites (HDMs). Conversely, it also suggested that frequent consumption of high plant-based protein diets has a significant protective association on subjects with AD and allergic backgrounds.

During inflammation and tissue injury, it was recommended to have a higher protein intake than the usual requirement (10–35% calorie needs in a normal adult aged ≥18 years) (Guadagni and Biolo, 2009; Wolfe et al., 2017). However, protein consumption has been reported to exceed the recommended daily calorie needs in developed nations and can impose detrimental health effects (Bilsborough and Mann, 2006). Even in less developed countries, a dietary pattern characterized by a high protein, calorie, and saturated fat intake was found to be associated with metabolic syndrome among healthy adults (Atasi et al., 2022). Although findings from the Framingham Heart Study Offspring Cohort suggested that an increase in biomarkers of inflammation and oxidative stress was lower in those with higher protein intake, this was specific for plant protein and not animal protein (Hruby and Jacques, 2019). It was further indicated in a separate clinical trial with 20 patients with AD that a vegetarian diet improved skin inflammation by attenuating prostaglandin E_2_ synthesis in monocytes (Tanaka et al., 2001). Similarly, we showed that the type of protein source was important to impact the associated risks of disease outcomes.

The effects of high animal protein consumption on systemic inflammation are evident in several studies. A randomized, acute clinical intervention study demonstrated an upregulation of CCL5 in the postprandial blood serum after meals supplemented with animal proteins (Holmer-Jensen et al., 2011). In addition, diets high in chicken proteins were suggested to affect neuroinflammation by increasing IL-1β, IL-6, and TNF-ɑ (Zhang et al., 2020). In contrast, plant-based nutritional habits have been often attributed to decreased risks of chronic diseases associated with inflammation by lowering circulating inflammatory biomarkers and relieving inflammatory state (Craddock et al., 2019). In support of this view, an intervention trial revealed that high plant protein diets for 6 weeks lowered the expression of *TGF-β1* and proinflammatory adipokines (chemerin and progranulin) in blood serum (Markova et al., 2020). Besides its immunomodulatory potential, TGF-β1 has a key role in wound epithelialization and skin homeostasis (Ramirez et al., 2014). A high exposure level of TGF-β1 in breast milk during the first month of life has been correlated with eczema later in life (Morita et al., 2018). Specifically, the TGF-β1–SMAD3 signaling pathway has been highlighted in AD murine models to influence the production of systemic IgE and promote allergen-induced skin inflammation (Anthoni et al., 2007). These results may partly explain the strongly associated protection between a frequent intake of high plant-based protein foods and AD.

In addition, the increased intake of mammal-derived protein-rich foods may lead to a change in the overall nutrition composition profiles, especially in the levels of total saturated fatty acids. Saturated fatty acids have been reported to be positively associated with increased prevalence of atopic diseases in developed countries (Black and Sharpe, 1997). High saturated fatty acids in diets were suggested to promote IgE synthesis and result in low-grade chronic systemic inflammation (Milanski et al., 2009). Data from the 2015 Canadian Community Health Survey - Nutrition highlighted that a dietary pattern of plant-based foods avoided excessive energy intake compared with red/processed meat dishes and minimized high saturated fatty acids, total cholesterol, and low-density lipoprotein cholesterol (Shafiee et al., 2022). Given the multiple benefits of plant-based foods to optimize human health, many international dietary guidelines encourage the incorporation of plant-based foods in diets (Liu, 2013; Rodriguez-Casado, 2016) As part of Singapore’s national efforts to promote healthy dietary habits, the Health Promotion Board recommended a mixture of animal-based and plant-based proteins from whole, fresh foods in meals to obtain a good source of essential nutrients and a healthy fat content (Health Hub, 2022).

Although we had examined the same association between various protein intakes and IE, there were no significant associations. This is consistent with a previous study in mice with psoriasis-like conditions whereby the feeding of high-protein diets exacerbated systemic inflammatory responses but had little effect on cutaneous conditions (Knopp et al., 2021). These results corroborated the idea that a frequent intake of high-protein foods in diets promoted the associated risks of AD by manifesting on top of the atopy predisposition. Thus, the influence of dietary proteins might be stronger in those with an allergic background and cutaneous hypersensitivity than in those without. Because AD forms a critical part of the classic triad of atopy, frequent intake of high-protein foods in diets may also influence and promote associated risks of other atopic diseases (Helm, 2004; Paller et al., 2019). More studies should be conducted to thoroughly assess the association between a diet of high protein intake and the development of other atopic diseases such as allergic rhinitis and asthma in addition to AD.

There is increasing evidence to suggest that dietary factors can affect microbiota diversity and composition to reduce the risk of AD development and severity in early life (Abrahamsson et al., 2012; Liu et al., 2022). In addition, studies highlighted that the source, concentration, and amino acid balance of dietary proteins drive the composition and function of gut microbes (Zhao et al., 2019). Protein digestion in the gut producing metabolites such as ammonia, amines, short-chain fatty acids, and hydrogen sulfide may trigger proinflammatory signaling pathways in epithelial cells and potentially contribute to AD pathogenesis (Abdallah et al., 2020). Apart from providing plant-based protein, a plant-based diet is rich in fibers, which act as a substrate to encourage anti-inflammatory effects and diverse growth of beneficial bacteria (Tomova et al., 2019). More studies are necessary to elucidate the specific mechanisms of gut microbiota in influencing AD with regard to plant-based food intake. Recent research has proposed targeting the gut microbiota on immune responses as a personalized dietary approach to improve AD symptoms (Alam et al., 2022).

One of the main study limitations lies within its cross-sectional design, which cannot be used to determine the causal direction between dietary habits of protein-based diets and AD. Furthermore, we cannot reveal the temporal sequence because the study was conducted retrospectively. There was also a lack of detailed information on cooking methods and a limited variety of food types in our dietary measurement. However, this study included a sufficiently large population with a high participant response rate, and the dietary indices used were carefully designed with established criteria and validated guidelines. Although there are concerns of potential recall bias in reporting diet, the effect of individual measurement errors is diluted, and the overall estimate of dietary intake frequencies is likely to be accurate with a large sample. Moreover, questionnaires were directly administered to subjects, and they were able to clarify doubts to reduce misunderstandings and errors. Although we have controlled for certain prominent confounding factors, including parental eczema, to address the genetic predisposition, we acknowledged that there might be other genetic and environmental factors not fully accounted for in our cross-sectional study. Therefore, prospective cohort studies can be conducted to provide valuable insights into the long-term relationships between dietary protein intake and AD/IE. Genetic and environmental information can be collected and controlled from subjects at baseline and throughout the follow-up period. This enables a more comprehensive assessment of gene–environment interactions that can help identify subgroups that may be more susceptible to the effects of dietary protein intake and better isolate the true association between dietary protein intake and AD/IE. At this stage, our findings provide strong initial evidence to improve the understanding of the relationship between dietary protein intake and AD/IE. Future research should be undertaken to thoroughly investigate the reliability of our dietary indices by cross-validating them in other independent cohorts but with more rigorous measures of dietary intake. This can involve the use of a detailed food frequency questionnaire that is more culturally based, in terms of food types/amount, culinary use, and nutritional compositions. Visual aids relating to standard portion sizes can also be employed to improve participants’ responses. By doing so, we seek to contextualize our results in relation to established metrics for chronic diseases such as the Alternative Healthy Eating Index, which holds significant importance in public health research (Morze et al., 2020).

Overall, the results in this study broadly support the work of other studies in this area linking dietary habits with chronic diseases and corroborate the ideas of how animal-based protein in diets may impose a possible detrimental risk to disease development, whereas plant-based protein may have associative protection against diseases. These data provide some valuable insights into how plant-based protein diets can be considered for nutritional treatment and clinical intervention in those with an allergic background and AD disease.

## Materials and Methods

### Study cohort

A cross-sectional study was conducted from 2005 to 2019 to investigate the epidemiology of allergy and atopic disease. A nonbias and random volunteer sample of staff and students was consecutively taken from universities in both Singapore (National University of Singapore, Singapore, Singapore) and Malaysia (Universiti Tunku Abdul Rahman, Perak, Malaysia and Sunway University, Selangor, Malaysia). Data on the personal and family symptomatic histories for eczema, lifestyles, and dietary habits were collected through an investigator-administered questionnaire that was validated by the International Study of Asthma and Allergy in Childhood protocol. The overall combined cohort of the Singapore/Malaysia Cross-sectional Genetic Epidemiology Study consists of 16,336 subjects. We first excluded 2,547 subjects who had missing responses for dietary habits, age, and sex. Owing to the unequal percentages of the three main races in Singapore and Malaysia, there was a lower probability of sampling among other ethnic groups, and the results obtained from the epidemiological investigation might not be fully representative of these ethnic groups (Department of Statistics Singapore, 2020). Thus, we have selected the major ethnic group Chinese (n = 11,494) to minimize ascertainment bias and the loss of statistical empowerment in our analysis (Figure 1).

### Disease definitions

As a genetic predisposition toward the development of immediate hypersensitivity against environmental allergens, allergic sensitization can be assessed experimentally by the presence of allergen-specific IgE or positive SPT to common allergens (Cohen et al., 2003). Among the local HDM fauna, *Blomia tropicalis* and *Dermatophagoides pteronyssinus* were found to be the most common yet important source of allergens in Singapore and the tropics (Chew et al., 1999a, 1999b). Past allergen studies have established that IgE sensitization was highly associated with HDM allergens, with >80% of individuals being HDM serum IgE positive (Andiappan et al., 2014). Thus, we objectively assessed all 11,494 Chinese subjects for their allergic sensitization status through an SPT for their reactivity to common HDMs, *B. tropicalis* and *D. pteronyssinus*. Subjects who had taken antihistamines or any related allergy medications within 3 days before the study were declined to ensure reliability in SPT. Those who tested positive for either one of the HDMs will have a wheal diameter ≥3 mm compared with the negative saline control. Subjects who were SPT negative did not show any elevation of allergen-specific IgE and did not have a wheal diameter ≥3 mm when tested with either HDM.

In this study, skin disease was historically defined. Per validated guidelines from the United Kingdom Working Party Criteria (Williams et al., 1994) and Hanifin and Rajka Criteria (Hanifin and Rajka, 1980), we defined eczema symptoms to be having a recurrent itchy rash for at least 6 months that was in the flexural regions, including under the buttocks and behind the neck, cheeks, ears, or eyes. During the collection, our trained personnel also concurrently assessed for the presence of an itchy rash. The assessments by our trained personnel were cross-validated periodically by a dermatologist and found to be concordant. The incorporation of SPT to HDMs alongside symptomatic histories in defining AD showed high sensitivity and specificity in a previous study compared with the clinical standard of having allergic comorbidities (Lim et al., 2022). For the purpose of this study, AD cases were defined as having eczema symptoms and a positive SPT response as described earlier. IE cases were defined as having eczema symptoms and an SPT-negative response (Brenninkmeijer et al., 2008; Karimkhani et al., 2015). Nonallergic noneczema control subjects were defined by not having eczema symptoms and a negative SPT response. Figure 1 shows the population distribution of SPT reactivity, eczema symptoms, and disease cases and controls. For more information, refer to Lim et al. (2022) on the Singapore/Malaysia Cross-sectional Genetic Epidemiology Study cohort demographics and various AD phenotypes.

### Dietary intake assessment and dietary indices

A section adapted from the International Study of Asthma and Allergy in Childhood phase III study was included to assess the dietary habits of 16 food types over the past 12 months (Ellwood et al., 2005). We asked the question “In the past 12 months, how often, on average, did you eat or drink the following?” for meat (e.g., beef, lamb, chicken, or pork), seafood (including fish), fruits, vegetables (green and root), pulses (pea, beans, lentils), cereals (including bread), pasta, rice, butter, margarine, nuts, potatoes, milk, egg, burgers/fast food, and yakult/vitagen/similar yogurt drinks. Subjects reported their intake frequencies as either “never or only occasionally,” “once or twice per week,” or “most or all days.”

**Figure 4:**
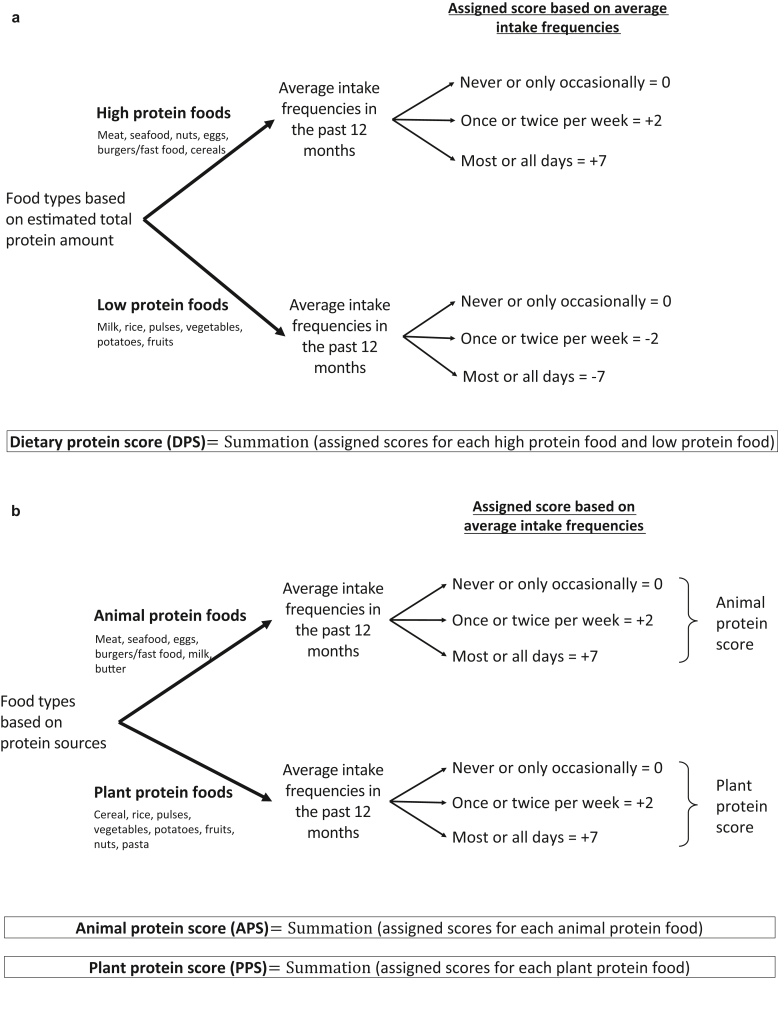
**Flowchart illustrating the design of various dietary indices.** (**a**) Dietary protein score to estimate the total protein intake in diets, (**b**) animal protein score and plant protein score to estimate the animal and plant-based protein intake in diets, respectively.

On the basis of the intake frequency of selected food types, a specific score was assigned to the corresponding intake frequencies—0 (never or only occasionally), 2 (once or twice per week), and 7 (most or all days)—following the validated rubric by Manousos et al. (1983). We first derived DPS to assess the total protein intake in diets. A total of 12 food types were categorized according to their total estimated protein amount, for a usual serving size of 100 g per edible portion, from the nutrient composition database by the Health Promotion Board (Singapore, Singapore) (Tables 16 and 17). Food types with high total estimated protein comprised meat, seafood, nuts, eggs, burgers/fast food, and cereals, whereas food types with low total estimated protein comprised milk, rice, pulses, vegetables, potatoes, and fruits. A positive score was prepended to high-protein foods, whereas a negative score was prepended to low-protein foods. The summation of assigned scores for all 12 food types resulted in DPS (Figure 4a). A one-of-three cut off was selected to categorize subjects into their respective groups for all dietary indices. Adequate sensitivity analyses based on the preliminary population were performed to ensure robustness and reliability in designing the dietary indices and choosing the cut offs. A total of 3,941 subjects were grouped into low DPS (score ≤ −6); 4,588 were grouped into moderate DPS (score −5 to 0); and 2,965 were grouped into high DPS (score >0). A subject with a low DPS represents a low intake frequency of high-protein foods (i.e., frequently consumes more low-protein foods than high-protein foods) and vice versa.

**Table 16:** Information on the Estimated Total Protein Amount, Based on the Usual Serving Size for the 16 Food Types, Retrieved from the HPB Database: The Average Total Protein Amount (Grams per 100 g Edible Portion) for Each Food Type

Food Types Analyzed	Grams per 100 g Edible Portion
High protein foods	
Meat (e.g., beef, lamb, chicken, pork)	25.06
Seafood (including fish)	22.73
Nuts	21.40
Eggs	12.20
Burgers/fast food	10.50
Cereals (including bread)	9.68
Low protein foods	
Pasta	6.60
Milk	3.97
Rice	2.56
Pulses (peas, beans, lentils)	2.48
Vegetables (green and root)	2.15
Potatoes	1.98
Margarine	1.20
Fruits	0.65
Butter	0.65
Probiotic drinks	0.35

Abbreviation: HPB, Singapore Health Promotion Board.

The estimated total protein amount was used as a reference to categorize the 16 food types into high-protein foods and low-protein foods. Information on estimated total protein amount (grams per 100 g edible portion) was retrieved from the Singapore HPB database at https://focos.hpb.gov.sg/eservices/ENCF/. The data were accessed in September 2022. The estimated total protein amount values were calculated on the basis of the list of common foods for each food type.

**Table 17:** Information on the Estimated Total Protein Amount, Based on the Usual Serving Size for the 16 Food Types, Retrieved from the Singapore HPB Database: List of Specific Foods Adopted for the Averaging of Total Protein Amount (Grams per 100 g Edible Portion) for Each Food Type

List of Common Foods Adopted for Each Food Type	Grams per 100 g Edible Portion
Animal-based protein foods	
Meat (e.g., beef, lamb, chicken, pork)	
Beef, boneless, unspecified cut, cooked, lean only	31.3
Pork (pork, boneless, unspecified cut, cooked, lean, and fat)	25.5
Chicken (chicken, broilers or fryers, drumstick, meat and skin, cooked, stewed)	25.3
Lamb (mutton cooked)	23.2
Beef, boneless, unspecified cut, raw, lean and fat	20.0
Seafood (including fish)	
Cuttlefish, cooked	32.5
Prawn, king, cooked	23.7
Steamed fish, unspecified	20.2
Crab, freshwater, boiled	19.2
Molluscs, scallop, mixed species, cooked, breaded, and fried	18.1
Eggs	
Egg, hen, whole, raw	12.9
Egg, duck, whole, raw	12.6
Egg, goose, whole, raw	11.1
Burgers/fast food	
Drumstick, original recipe, KFC	26.0
Winglets, KFC	14.0
Hamburger, Burger King	14.0
Hawaiian 12” Large Pan Pizza, Pizzahut	13.0
McSpicy, Mcdonald’s	11.0
Fillet-o-fish, McDonald’s	10.0
McChicken, McDonald’s	8.0
Onion rings, small, Burger King	4.0
KFC cheese fries	3.0
Apple pie, McDonald’s	2.0
Milk	
Milk, whole, evaporated, canned	7.6
Low-fat milk	3.9
UHT chocolate milk	3.3
Full cream milk	3.2
Soya bean milk, with sugar (hawker centre)	3.2
Milk, white	3.2
Butter	
Butter, salted	0.7
Butter, unsalted	0.6
Plant-based protein foods	
Nuts	
Nuts, peanut, with shell, dry roasted	29.0
Nuts, almonds	21.3
Nuts, cashew nut, raw	21.2
Nuts, pistachio nuts, raw	20.6
Nuts, hazelnuts	15.0
Cereals (including bread)	
Bread, French	11.8
Bread, brown	9.7
Bread, white	9.7
Breakfast cereal, plain, contains whole grains	7.5
Pasta	
Pasta, egg, dried raw	12.7
Pasta, marinara	9.5
Creamy chicken pasta	6.5
Meatball pasta	6.3
Plain Pesto pasta	6.0
Pasta, wholemeal, boiled	5.8
Crayfish pasta	5.6
Noodles, instant, dry, with seasoning, cooked	5.2
Pasta, white, boiled	4.2
Creamy mushroom pasta	4.2
Rice	
Brown rice cooked	3.1
White rice cooked	2.8
Rice, glutinous, white, cooked	2.0
Pulses (peas, beans, lentils)	
Long beans, boiled	2.5
Snow peas, raw	2.5
Beans, French, raw	2.4
Vegetables (green and root)	
Vegetables, dark green non-leafy, unspecified, boiled	2.8
Onions, ginger, garlic	2.5
Vegetable, dark green leafy, unspecified, boiled, drained	2.2
Carrot, boiled	1.1
Potatoes	
Potato, old, baked, flesh and skin	2.3
Sweet potato, boiled, without skin	1.7
Fruits	
Banana, raw, unspecified type	1.1
Orange	1.0
Watermelon	0.8
Papaya	0.4
Pear, green, raw with peel	0.3
Apples, raw, without skin	0.3
Others	
Probiotic drinks	
Yogurt drink, flavored, nonfat	1.2
Margarine	
Margarine spread, polysaturated	0.4
Margarine spread, monounsaturated	0.4
Margarine spread, polyunsaturated, reduced fat, reduced salt	0.3
Margarine spread, monounsaturated, reduced fat, reduced salt	0.3

Abbreviations: HPB, Singapore Health Promotion Board; KFC, Kentucky Fried Chicken; UHT, ultra heat treatment.

The estimated total protein amount was used as a reference to categorize the 16 food types into high-protein foods and low-protein foods. Information on estimated total protein amount (grams per 100 g edible portion) was retrieved from the Singapore HPB database at https://focos.hpb.gov.sg/eservices/ENCF/. The data were accessed in September 2022. The estimated total protein amount values were calculated on the basis of the list of common foods for each food type.

Next, we derived APS and PPS to assess the intake frequency of animal-derived protein and plant-derived protein in diets, respectively. Animal-derived protein foods were meat, seafood, milk, eggs, burgers/fast food, and butter, whereas plant-derived protein foods were cereals, rice, pulses, vegetables, potatoes, fruits, nuts, and pasta. For APS and PPS, only positive scores were allocated to their respective intake frequencies of various animal-derived protein and plant-derived protein foods. Similarly, the summation of the assigned score for animal food types yielded APS, and the summation of the assigned score for plant food types yielded PPS (Figure 4b). For APS, 3,588 subjects had a low APS (score <18); 3,875 had a moderate APS (score = 18–24); and 4,031 had a high APS (score > 24). For PPS, 3,551 subjects had a low PPS (score < 26); 3,883 subjects had a moderate PPS (score = 26–33); and 4,060 had a high PPS (score > 33). A low APS and PPS represent low intake frequency of animal-derived protein foods and plant-derived protein foods, respectively. Information on the design of various dietary indices is presented in Figure 4. We have previously described the use of a dietary fat score (diet quality based on dietary fat score) (Lim et al., 2022) and quality of diet based on GI score (Say et al., 2022) to assess the intake of fat and GI, respectively. In this study, we evaluated these scores in combination with the protein scores to determine the various associated risks for AD and IE. Kindly refer to the earlier studies for more information on the computation of diet quality based on dietary fat score and quality of diet based on GI score.

### Statistical analysis

Logistic regression models were conducted to analyze the association between dietary indices and various outcomes. Outcomes of logistic regression were presented in ORs, 95% confidence intervals, and a *P*-value. The statistical significance of the results was defined as logistic regression *P* < 0.003 (as adjusted by Bonferroni’s correction for multiple comparisons), and the OR range did not cross 1.000. All statistical analyses and drawing of balloon plots were performed using R program, version 2021.09.0.351 (http://www.rstudio.com), whereas Microsoft Excel (http://office.microsoft.com/en-us/excel/) was used for data entry and drawing of bar plots. A chi-square test was performed to determine whether the distribution of a given categorical variable was different across different outcome groups. A chi-square *P <* 0.001 (as adjusted by Bonferroni’s correction) was considered statistically significant. SF is a statistical method to assess the potential binary interactions between risk factors in case-control studies, especially for study of complex diseases (Cortina-Borja et al., 2009). SF analysis is easy to use and clear to interpret. In SF analysis, we examine whether the combined effects of animal-derived and plant-derived protein intake in diets exhibit synergy (greater than additive effect) or antagonism (lesser than additive effects) in influencing the susceptibility of skin diseases. For a detailed explanation of SF methodology, interested readers can refer to the original work by Cortina-Borja et al. (2009). Understanding antagonistic interactions provides insights into the complex interplay between dietary proteins (of different sources) and potentially guides the development of dietary interventions in the future.

### Ethics statement

This study was conducted in accordance with the principles of the Declaration of Helsinki and Good Clinical Practices and in compliance with local regulatory requirements. Ethical approval was granted from the Institutional Review Board of the National University of Singapore (Singapore, Singapore) (National University of Singapore Institutional Review Board reference codes NUS-07-023, NUS-09-256, NUS-10-445, NUS-13-075, NUS-14-150, and NUS-18-036), the Scientific and Ethical Review Committee of Universiti Tunku Abdul Rahman (reference code U/SERC/03/2016), and Sunway University Research Ethics Committee (reference code SUREC 2019/029). Informed consent was obtained from all subjects before the study through a signed consent form. Parental consent was obtained for subjects below the age of 18 years.

### Data availability statement

All data used and included in this study are available from the corresponding author (FTC).

## ORCIDs

Jun Jie Lim: http://orcid.org/0000-0001-5485-1749

Kavita Reginald: http://orcid.org/0000-0003-1530-5934

Yee-How Say: http://orcid.org/0000-0003-2363-5239

Mei Hui Liu: http://orcid.org/0000-0001-7405-7008

Fook Tim Chew: http://orcid.org/0000-0003-1337-5146

### Disclaimer

The funding agencies had no role in the study design, data collection and analysis, decision to publish, or preparation of the manuscript.

## Conflict of Interest

FTC reports grants from the Singapore Ministry of Education Academic Research Fund, Singapore Immunology Network, National Medical Research Council, Biomedical Research Council, National Research Foundation, Singapore Food Agency, and the Agency for Science Technology and Research during the conduct of the study and has received consultancy fees from Sime Darby Technology Centre, First Resources, Genting Plantation, Olam International, and Syngenta Crop Protection, outside the submitted work. The remaining authors state no conflict of interest.
